# Supplementary material for: Upconversion Luminescence Response of a Single YVO4:Yb, Er Particle
Source: Micromachines (Basel). 2023 May 19;14(5):1075. doi: 10.3390/mi14051075 (PMC10221738; doi:10.3390/mi14051075)
Supplement: Supplementary file 1 [file micromachines-14-01075-s001.zip › micromachines-2396116-Supplementary Material.pdf]

Supplementary Material

Upconversion luminescence response of a single YVO_4:Yb, Er particle

D.K. Zharkov, A.V. Leontyev, A.G. Shmelev, L.A. Nurtdinova, A.P. Chuklanov, N.I. Nurgazizov, V.G. Nikiforov*

Zavoisky Physical-Technical Institute, FRC Kazan Scientific Center of RAS, Sibirsky tract, 10/7, 420029, Kazan, Russia

[*] *E-mail address*: vgnik@mail.ru

Element	Weigth %	Atomic %
C K	3.04	5.87
O K	38.73	56.17
F K	7.14	8.72
Na K	0.69	0.70
Si K	28.21	23.31
Ni K	8.58	3.39
Er M	3.60	0.50
Yb M	10.01	1.34
Sum	100.00	

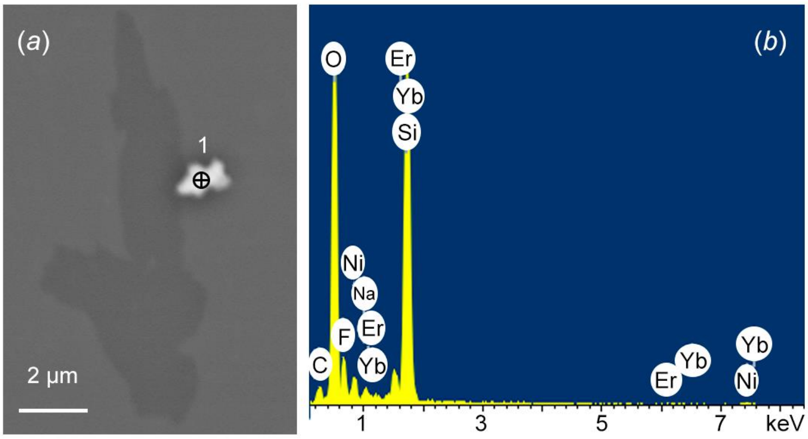

Fig. S1. SEM image (a) and EDS spectrum (b) of the YVO_4:Yb, Er SP.

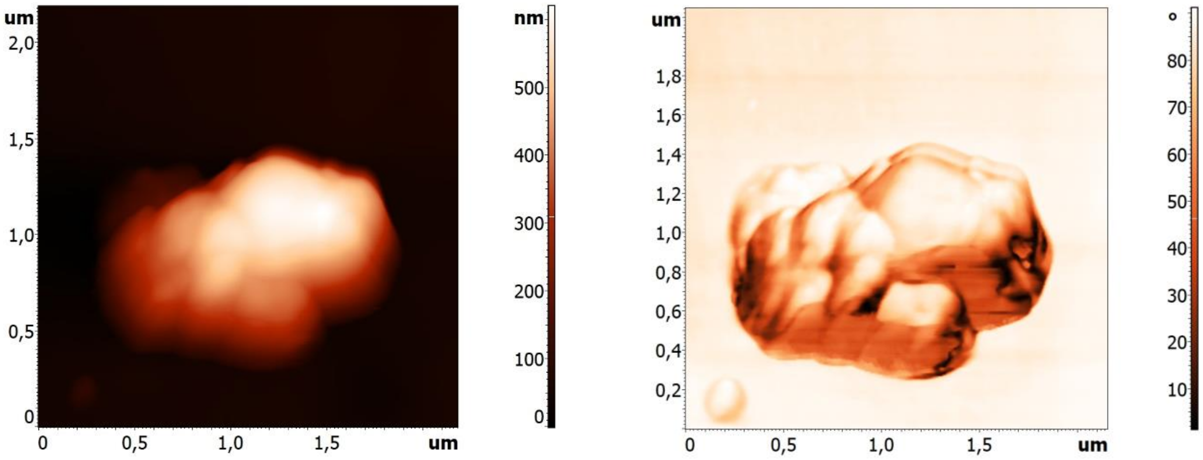

Fig. S2. AFM image (left) and phase-contrast image (right) of the YVO_4:Yb, Er SP.

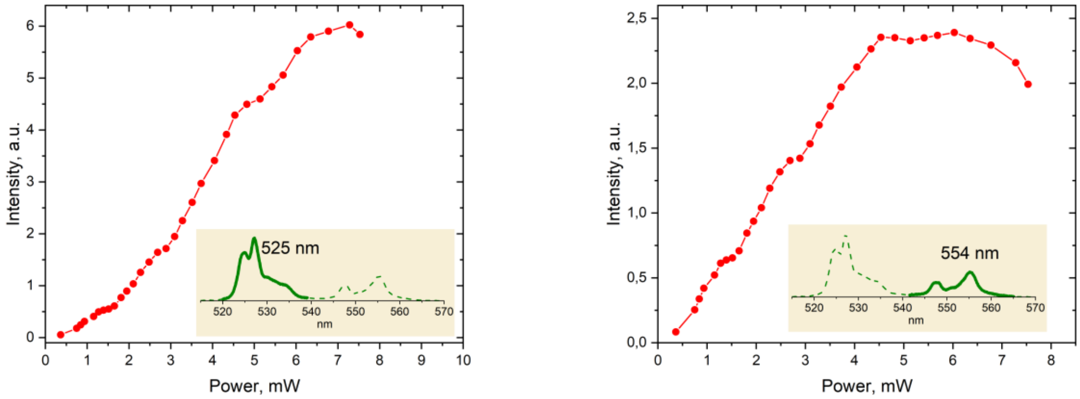

Fig. S3. Dependences of the integral intensities of Er^{3+} luminescence in the 520-540 nm (left panel) and 540-565 nm (right panel) ranges on the laser power for YVO_4: Yb, Er SP. Despite the Fig. 7, plots are presented in linear-linear format.
